# Different adiposity indices and their associations with hypertension among Chinese population from Jiangxi province

**DOI:** 10.1186/s12872-020-01388-2

**Published:** 2020-03-05

**Authors:** Lihua Hu, Guiping Hu, Xiao Huang, Wei Zhou, Chunjiao You, Juxiang Li, Ping Li, Yanqing Wu, Qinghua Wu, Zengwu Wang, Runlin Gao, Huihui Bao, Xiaoshu Cheng

**Affiliations:** 1 Department of Cardiovascular Medicine, the Second Affiliated Hospital of Nanchang University, No. 1 Minde Road, Nanchang of Jiangxi, 330006 China; 2 School of Medical Science and Engineering, Beihang University, Beijing, China; 3 Center for Prevention and Treatment of Cardiovascular Diseases, the Second Affiliated Hospital of Nanchang University, Nanchang of Jiangxi, China; 4 Division of Prevention and Community Health, National Center for Cardiovascular Disease, Fuwai Hospital, Peking Union Medical College & Chinese Academy of Medical Sciences, Beijing, China; 5 Fuwai Hospital, Peking Union Medical College & Chinese Academy of Medical Sciences, Beijing, China

**Keywords:** Hypertension, Waist-to-height ratio, Visceral adipose index, Body fat percentage, Receiver operating characteristic curve

## Abstract

**Background:**

To date, the best adiposity index that predicts or associates strongly with hypertension remains controversial. Therefore, we aimed to compare the performance of different adiposity indices [BMI (body mass index), WC (waist circumference), WHtR (waist-to-height ratio), ABSI (a body shape index), VAI (visceral adipose index), BFP (body fat percentage)] as associates and potential predictors of risk of hypertension among Chinese population.

**Methods:**

A cross-sectional survey was conducted in Jiangxi province, China from 2013 to 2014. A total of 14,573 participants were included in the study. The physical measurements included body height, weight, WC, BFP and VAI. Multivariate logistic regression analysis was performed to assess the associations between different adiposity indices and the prevalence of hypertension. Receiver operating characteristic (ROC) analysis was also performed.

**Results:**

All adiposity indices were independently and positively associated with the prevalence of hypertension in a dose response fashion. The area under the curves (AUCs) for WHtR, BFP and VAI were significantly larger than those for other adiposity indices in both males and females (all *P* < 0.01). For males, no statistically significant difference was found in AUCs among WHtR and BFP (0.653 vs. 0.647, *P* = 0.4774). The AUC of WHtR was significantly higher than VAI (0.653 vs. 0.636, *P* < 0.01). For females, the AUCs demonstrated that WHtR was significantly more powerful than BFP and VAI (both *P* < 0.05) for predicting hypertension [WHtR, 0.689 (0.677–0.702); BFP, 0.677 (0.664–0.690); VAI, 0.668 (0.655–0.680)]. Whereas no significant differences were found in AUCs for hypertension among BFP and VAI in both sexes (all *P* > 0.1). The AUCs for hypertension associated with each adiposity index declined with age in both males and females. For subjects aged < 65 years, WHtR still had the largest AUC. However, for participants aged ≥65 years, BMI had the largest AUC.

**Conclusion:**

The findings indicated that WHtR was the best for predicting hypertension, followed by BFP and VAI, especially in younger population.

## Background

Hypertension is increasingly regarded as a widespread global disease and the leading modifiable risk factor for cardiovascular disease [1, 2]. A number of proven, highly effective, and well tolerated lifestyle and drug treatment strategies can lower blood pressure (BP). Despite this, the prevalence of hypertension is still high. Recently, the results from China Hypertension Survey during 2012–2015 showed that the prevalence of hypertension among the Chinese adult population was 23.2% [3]. Currently, it is estimated that more than 2.4 billion individuals in China suffer from hypertension. Previous studies have reported that the modifiable risk factors for hypertension include salt intake, obesity, abdominal obesity, smoking, drinking and sleep duration [4–6].

With the improvement of people’s living standard and life rhythm speeding up, obesity has also become a growing global public health problem. Previous studies showed that obesity and abdominal obesity were considered risk factors for multiple chronic diseases, including diabetes, hypercholesterolemia, asthma, cancer, cardiovascular disease (CVD) and hypertension [7, 8]. To date, body mass index (BMI) and waist circumference (WC) are still promulgated as the main epidemiological measures of obesity and abdominal obesity [6]. However, their usefulness suffer from their inability to account for body adipose distribution [9, 10]. Differences in adipose tissue distribution may contribute to the heterogeneity of clinical and biological manifestations of obesity [11]. Some anthropometric indices have been developed to specifically describe body fat distribution, including waist-to-height ratio (WHtR), a body shape index (ABSI), visceral adipose index (VAI) and body fat percentage (BFP). Some studies reported that WHtR was a better predictor of hypertension, diabetes, and hyperlipidemia than BMI and WC [12, 13]. Body shape, as measured by ABSI, was a substantial risk factor for premature mortality in the general population derivable from basic clinical measurements [14]. VAI was located in the abdomen and intra-abdominal contents, not the subcutaneous fat abundant in the buttocks and lower limbs. Several studies showed that VAI was superior to BMI and WC in predicting hypertension [5, 15]. BFP was calculated as the total mass of fat divided by total body mass. Previous studies indicated that BFP was positively associated with risk of hypertension, SBP and DBP levels [16].

However, the best adiposity index that predicts or associates strongly with hypertension remains controversial and inconclusive. Few studies address the associations between the six adiposity indices and hypertension. Therefore, the aims of this study were to compare the performance of different adiposity indices as associates and potential predictors of risk of hypertension among Chinese population.

## Methods

### Study design and population

A detailed description of the study have been reported elsewhere in previous publications [4, 5, 17]. Briefly, the cross-sectional epidemiological investigation, a community based study, was conducted during November 2013 to August 2014 in Jiangxi province, China. It was part of the China Hypertension Survey encompassed 31 provinces and 262 countries [3]. Ethical approval was obtained from the ethics review boards of the Second Affiliated Hospital of Nanchang University and the Fuwai Cardiovascular Hospital (Beijing, China). Written informed consent was obtained from each participant. If individuals were younger than the age of 18, written informed consent was obtained from their parents or legal guardians.

As a result, a total of 15,296 participants completed the investigation [4, 5]. After excluding those with missing height value (*n* = 97), WC value (*n* = 34), VAI value (*n* = 570), and BFP value (*n* = 22), finally, a total of 14,573 participants were analyzed.

### Anthropometric and bioelectrical measurements

The methods of anthropometric and bioelectrical measurements have been reported in our previous publication [5]. Height was measured without shoes to the nearest 0.5 cm. WC was also measured to the nearest 0.5 cm midway between the lowest rib and the superior border of the iliac crest with a flexible anthropometric tape. Basic metabolism rate (BMR), BFP, VFI and body weight without heavy clothing were measured by bioelectrical impedance methods using Omron body fat and weight measurement device (V- BODY HBF-371, OMRON, Kyoto, Japan). All measurements were taken twice and the average of the 2 values was adopted.

BMI was calculated as weight (kg)/height (m) [2]. WHtR was calculated as WC (cm)/height (cm). ABSI (m^11/6^ kg^-2/3^) and its standard deviation score (SDS) were calculated using the following formula:

$$ ABSI=\frac{WC}{BM{I}^{2/3} Heigh{t}^{1/2}} $$

### BP measurement and definition of hypertension

BP was measured with OMRON Professional PorTable Blood Pressure Monitor (HBP-1300, OMRON, Kyoto, Japan) three times on the right arm positioned at heart level after the participant was sitting at rest for 5 min, with 30 s between each measurement with an observer present. Then systolic BP (SBP) and diastolic BP (DBP) were calculated as the mean of three independent measures. According to 2010 Chinese guidelines for the management of hypertension, hypertension was defined as SBP ≥140 mmHg and/or DBP ≥90 mmHg, and use of antihypertensive medications within 2 weeks [18].

### Statistical analysis

Data are presented as mean ± standard deviation (SD) for continuous variables and as frequency (%) for categorical variables. Baseline characteristics of study population were described by sex. Comparisons between different sex groups were performed using chi-square tests for categorical variables and using two-sample t tests for continuous variables. The association between each adiposity index and the prevalence of hypertension was examined as a continuous variable per SD increment and also as a categorical variable using quartiles with the lowest quartile (Q1) as the reference group. Multivariate logistic regression analysis was performed to assess the odds ratios (ORs) and 95% confidence intervals (CI) for the associations between different adiposity indices (BMI, WC, WHtR, ABSI, BFP and VAI) and hypertension stratified by sex and age. Multivariable models were constructed as follows: model I was adjusted for age and sex; model II was further adjusted for area, smoking, drinking, education status, occupation, family history of hypertension, antihypertensive medications, sleep duration (workdays and non-workdays), BMR and RHR.

Receiver operating characteristic (ROC) analysis was performed to compare the performance of different adiposity indices as potential predictors of hypertension in both males and females. Areas under the ROC curves (AUCs) of these adiposity indices (BMI, WC, WHtR, ABSI, BFP, VAI and BMI combined with WC) were used as measure of predictive power of hypertension; statistical significance of the difference among them determined by applying the method of DeLong [19] et al. (1988) using MedCalc version 10.1.6.0 (MedCalc Software, Ostend, Belgium).

We also did the sensitivity analysis to ensure the robustness of results. ROC analysis was also used to compare the performance of adiposity indices as potential predictors of hypertension among participants without taking antihypertensive medications.

All data was established using Epi Data 3.02 software. After alignment correction, statistical analysis was performed using the statistical package R (http://www.r-project.org) and Empower (R) (www.empowerstats.com; X&Y Solutions, Inc., Boston, MA). A two side *P* value < 0.05 was considered to be statistically significant.

## Results

### Characteristics of the subjects

As shown in S1 Table and Table 1, a total of 14,573 participants (5961 males, and 9612 females) were included in this study with a mean age of 53.37 (17.63) years. Overall, the mean SBP and DBP levels were 125.84 ± 19.17 mmHg and 74.04 ± 10.61 mmHg, respectively. The prevalence of hypertension was 29.14% (4247/14573). The mean (SD) values for BMI, WC, WHtR, ABSI, BFP and VAI were 22.86 (3.65) kg/m^2^, 79.08 (9.65) cm, 0.50 (0.06), 0.0787 (0.0065) m^11/6^ kg^-2/3^, 27.42 (8.97) and 7.24 (4.28), respectively. Compared with females, males were more likely to have higher values in age, height, weight, WC, ABSI, VAI, BMR, SBP and DBP, to have lower values in WHtR and BFP, to have higher prevalence of hypertension, to be smokers, to be drinkers, to have higher educational level and to be employed (all *P* < 0.05). No significant differences were found between sex in terms of BMI, areas, antihypertensive medications or sleep duration (all *P* > 0.05).

**Table 1:** Baseline characteristics of study participants by sex

Characteristics^a^	Total (*N* = 14,573)	Male (*N* = 5961)	Female (*N* = 8612)	*P* value
Age, y	53.37 ± 17.63	53.80 ± 18.04	53.08 ± 17.34	0.014
Height, cm	157.08 ± 8.91	163.12 ± 7.73	152.90 ± 7.10	< 0.001
Weight, kg	56.51 ± 10.60	61.05 ± 11.07	53.36 ± 9.01	< 0.001
BMI, kg/m^2^	22.86 ± 3.65	22.92 ± 3.75	22.83 ± 3.58	0.136
WC, cm	79.08 ± 9.65	80.85 ± 9.69	77.85 ± 9.43	< 0.001
WHtR	0.50 ± 0.06	0.50 ± 0.06	0.51 ± 0.07	< 0.001
ABSI, m^11/6^ kg^-2/3^	0.0787 ± 0.0065	0.0788 ± 0.0062	0.0786 ± 0.0068	0.037
BFP	27.42 ± 8.97	23.42 ± 8.98	30.19 ± 7.85	< 0.001
VAI	7.24 ± 4.28	8.61 ± 4.65	6.29 ± 3.72	< 0.001
BMR, kcal	1256.46 ± 311.32	1387.86 ± 297.95	1165.52 ± 286.96	< 0.001
SBP, mm Hg	125.84 ± 19.17	127.80 ± 17.63	124.47 ± 20.06	< 0.001
DBP, mm Hg	74.04 ± 10.61	76.27 ± 10.47	72.48 ± 10.44	< 0.001
RHR, bpm	78.10 ± 11.20	77.41 ± 11.54	78.57 ± 10.93	< 0.001
Hypertension, n (%)	4247 (29.14)	1809 (30.35)	2438 (28.31)	0.008
Family history of hypertension, n (%)	3148 (22.44)	1195 (21.03)	1953 (23.40)	< 0.001
Urban, n (%)	7460 (51.19)	2855 (47.89)	4258 (49.44)	0.066
Current smokers, n (%)	2644 (18.19)	2489 (41.91)	155 (1.80)	< 0.001
Current drinkers, n (%)	3467 (23.87)	2344 (39.47)	1123 (13.08)	< 0.001
Education status, n (%)				< 0.001
≤ 6 years	7184 (49.87)	2337 (39.54)	4847 (57.06)	
6–12 years	6008 (41.71)	2942 (49.78)	3066 (36.09)	
> 12 years	1213 (8.42)	631 (10.68)	582 (6.85)	
Occupation, n (%)				< 0.001
Employed	5090 (35.27)	2347 (39.77)	2743 (32.16)	
Retired	1893 (13.12)	700 (11.86)	1193 (13.99)	
Unemployed	7448 (51.61)	2854 (48.36)	4594 (53.86)	
Antihypertensive medications, n (%)	1146 (7.86)	497 (8.34)	649 (7.54)	0.077
ACEI/ARB	308 (2.11)	140 (2.35)	168 (1.95)	0.101
CCB	813 (5.58)	353 (5.92)	460 (5.34)	0.133
Diuretics	15 (0.10)	9 (0.15)	6 (0.07)	0.132
β-Blockers	57 (0.39)	27 (0.45)	30 (0.35)	0.320
Others	127 (0.87)	54 (0.91)	73 (0.85)	0.710
Sleep duration, h				
Workdays	7.30 ± 1.44	7.29 ± 1.42	7.30 ± 1.46	0.877
Non-workdays	7.64 ± 1.45	7.62 ± 1.41	7.65 ± 1.48	0.190

*Abbreviations*: *BMI* Body mass index, *WC* Waist circumference, *WHtR* Waist-to-height ratio, *ABSI* A body shape index, *BFP* Body fat percentage, *VAI* Visceral adipose index, *BMR* Basal metabolism rate, *SBP* Systolic blood pressure, *DBP* Diastolic blood pressure, *RHR* Rest heart rate, *ACEI* Angiotensin-converting enzyme inhibitors, *ARB* Angiotensin II receptor blockers, *CCB* Calcium channel blockers

^a^Data are presented as number (%) or mean ± standard deviation

### Associations between different adiposity indices and the prevalence of hypertension

**Fig. 1:**
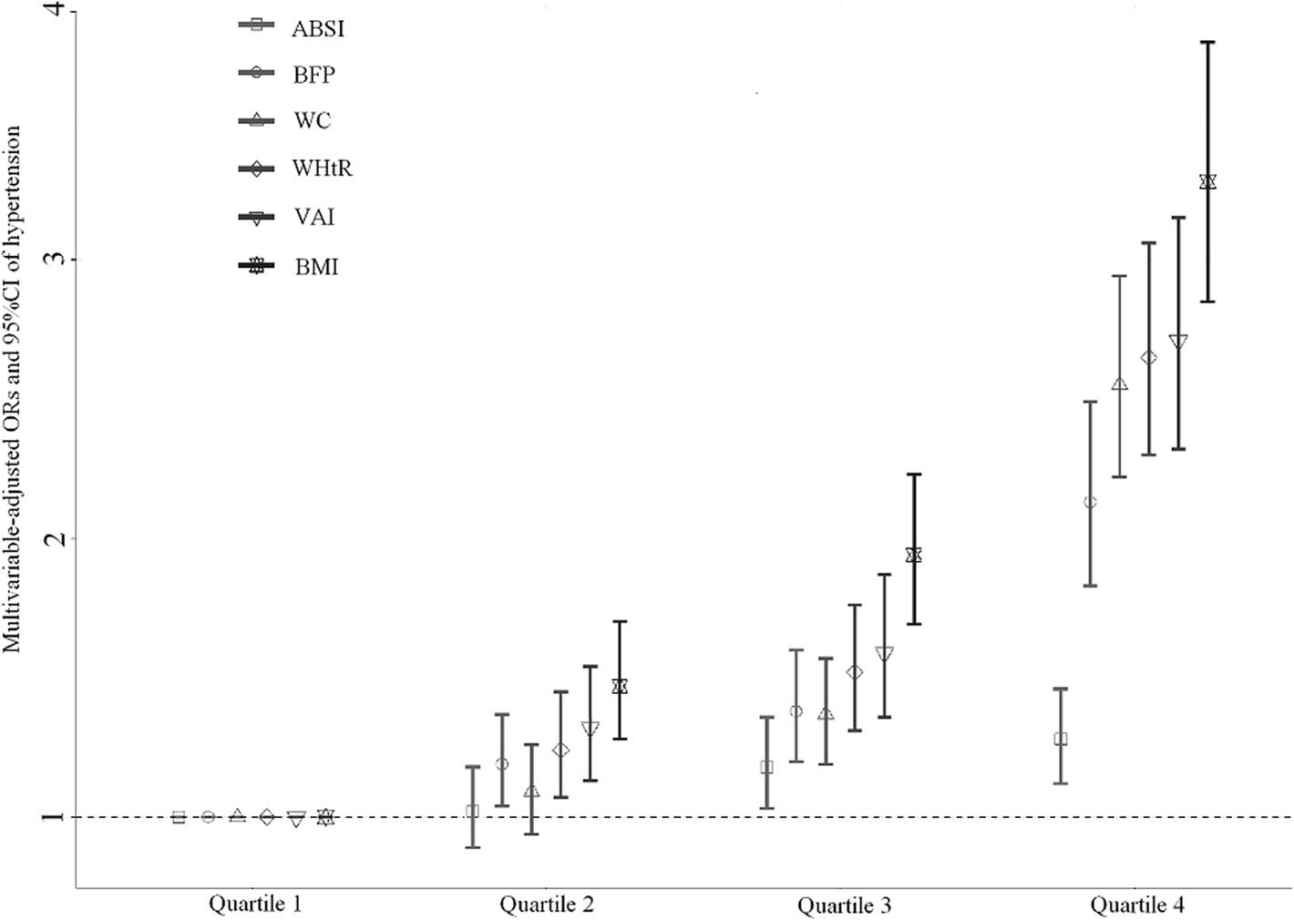
Multivariable-adjusted ORs (95%CI) of hypertension according to quartiles of BMI, WC, WHtR, ABSI, BFP and VAI. Adjusted for sex, age, area, smoking, drinking, education status, occupation, family history of hypertension, antihypertensive medications, sleep duration (workdays and non-workdays), BMR and RHR. Cut-points of quartiles:BMI (kg/m^2^) 20.30, 22.50, 25.00; WC (cm) 72.00, 78.00, 85.00; WHtR 0.46, 0.50, 0.54; ABSI (m^11/6^ kg-^2/3^) 0.0748, 0.0786, 0.0823; BFP 22.00, 27.00, 32.40; VAI 4.00, 7.00, 9.00

Figure 1 showed the multivariable-adjusted ORs and 95%CI for hypertension according to quartiles of six adiposity indices. Although ABSI and WC in Q2 were not significantly different from Q1 [ABSI: OR (95%CI) = 1.02 (0.89, 1.18), *P* = 0.740; WC: OR (95%CI) = 1.09 (0.94, 1.26), *P* = 0.246], we still observed a significant and progressive increase in the prevalence of hypertension with adiposity indices quartiles (all *P* for trend < 0.0001), suggesting a dose-dependent increase in prevalence of hypertension with all adiposity indices.

The associations between different adiposity indices based on z-score standardization and prevalence of hypertension were shown in Table 2. In fully adjusted model, BMI, WC, WHtR, ABSI, BFP and VAI were independently and positively associated with prevalence of hypertension (OR = 1.54, 1.52, 1.52, 1.05, 1.25 and 1.48, respectively, all *P* < 0.05). Subsequently, Table 3 shows the associations between six adiposity indices and hypertension stratified by sex and age. Each SD increment of adiposity indices except ABSI was significantly associated with higher prevalence of hypertension by sex and age (all *P* < 0.05). Moreover, the standardized ORs of adiposity indices except BFP in relation to hypertension tended to decrease with increasing age in both sexes.

**Table 2:** Standardized ORs and 95% CI for hypertension

	Hypertension OR (95%CI)		
	Crude model	Model I	Model II
BMI z-score	1.49 (1.43, 1.54)^***^	1.68 (1.61, 1.76)^***^	1.54 (1.47, 1.62)^***^
WC z-score	1.65 (1.59, 1.72)^***^	1.67 (1.60, 1.74)^***^	1.52 (1.45, 1.60)^***^
WHtR z-score	1.87 (1.80, 1.94)^***^	1.67 (1.60, 1.74)^***^	1.52 (1.45, 1.60)^***^
ABSI z-score	1.40 (1.34, 1.45)^***^	1.06 (1.02, 1.11)^**^	1.05 (1.00, 1.10)^*^
BFP z-score	1.44 (1.39, 1.50)^***^	1.33 (1.28, 1.39)^***^	1.25 (1.19, 1.32)^***^
VAI z-score	1.64 (1.58, 1.70)^***^	1.59 (1.52, 1.66)^***^	1.48 (1.41, 1.55)^***^

Model I: regression was done with adjustment for sex, age. Model II: regression was done with adjustment for sex, age, area, smoking, drinking, education status, occupation, family history of hypertension, antihypertensive medications, sleep duration (workdays and non-workdays), BMR and RHR

^*^P < 0.05, ^**^P < 0.01, ^***^P < 0.0001

**Table 3:** Standardized ORs and 95% CI for hypertension by sex and age

		Age groups (years)		
		15–44	45–64	≥65
Male				
BMI z-score	Crude OR	2.14 (1.84, 2.48)^***^	1.61 (1.46, 1.78)^***^	1.51 (1.36, 1.67)^***^
	Adjusted OR	1.92 (1.62, 2.28)^***^	1.52 (1.34, 1.72)^***^	1.38 (1.21, 1.56)^***^
WC z-score	Crude OR	2.34 (1.99, 2.75)^***^	1.71 (1.55, 1.88)^***^	1.43 (1.29, 1.57)^***^
	Adjusted OR	2.07 (1.72, 2.50)^***^	1.56 (1.38, 1.76)^***^	1.26 (1.11, 1.43)^**^
WHtR z-score	Crude OR	2.53 (2.11, 3.03)^***^	1.76 (1.58, 1.95)^***^	1.48 (1.33, 1.64)^***^
	Adjusted OR	2.09 (1.71, 2.56)^***^	1.55 (1.36, 1.76)^***^	1.32 (1.16, 1.50)^***^
ABSI z-score	Crude OR	1.28 (1.06, 1.54)^**^	1.23 (1.10, 1.37)^**^	0.99 (0.91, 1.08)
	Adjusted OR	1.09 (0.86, 1.38)	1.10 (0.97, 1.24)	0.96 (0.87, 1.06)
BFP z-score	Crude OR	1.22 (1.10, 1.35)^**^	1.34 (1.21, 1.47)^***^	1.41 (1.24, 1.61)^***^
	Adjusted OR	1.22 (1.07, 1.39)^**^	1.17 (1.05, 1.30)^**^	1.26 (1.10, 1.44)^**^
VAI z-score	Crude OR	1.89 (1.65, 2.17)^***^	1.52 (1.39, 1.66)^***^	1.41 (1.29, 1.53)^***^
	Adjusted OR	1.55 (1.32, 1.82)^***^	1.35 (1.21, 1.50)^***^	1.30 (1.16, 1.45)^***^
Female				
BMI z-score	Crude OR	2.08 (1.76, 2.46)^***^	1.52 (1.41, 1.65)^***^	1.49 (1.37, 1.62)^***^
	Adjusted OR	1.63 (1.32, 2.00)^***^	1.44 (1.32, 1.58)^***^	1.55 (1.40, 1.72)^***^
WC z-score	Crude OR	2.33 (1.94, 2.79)^***^	1.57 (1.45, 1.71)^***^	1.47 (1.35, 1.59)^***^
	Adjusted OR	1.66 (1.33, 2.07)^***^	1.44 (1.31, 1.58)^***^	1.48 (1.34, 1.64)^***^
WHtR z-score	Crude OR	2.50 (2.08, 2.99)^***^	1.59 (1.47, 1.73)^***^	1.49 (1.37, 1.61)^***^
	Adjusted OR	1.74 (1.40, 2.16)^***^	1.43 (1.30, 1.57)^***^	1.49 (1.36, 1.64)^***^
ABSI z-score	Crude OR	1.42 (1.17, 1.73)^**^	1.15 (1.06, 1.24)^*^	1.05 (0.98, 1.12)
	Adjusted OR	1.17 (0.93, 1.49)	1.05 (0.95, 1.16)	1.05 (0.97, 1.13)
BFP z-score	Crude OR	1.35 (1.19, 1.53)^***^	1.44 (1.31, 1.59)^***^	1.33 (1.21, 1.47)^***^
	Adjusted OR	1.28 (1.07, 1.52)^**^	1.27 (1.13, 1.42)^***^	1.30 (1.16, 1.46)^***^
VAI z-score	Crude OR	1.66 (1.43, 1.91)^***^	1.65 (1.50, 1.80)^***^	1.54 (1.39, 1.69)^***^
	Adjusted OR^a^	1.56 (1.28, 1.89)^***^	1.51 (1.36, 1.68)^***^	1.54 (1.37, 1.73)^***^

^a^Adjusted for age, area, smoking, drinking, education status, occupation, family history of hypertension, antihypertensive medications, sleep duration (workdays and non-workdays), BMR and RHR

^*^P < 0.05, ^**^P < 0.01, ^***^P < 0.0001

### Analysis of the predictive power of each index for hypertension

**Fig. 2:**
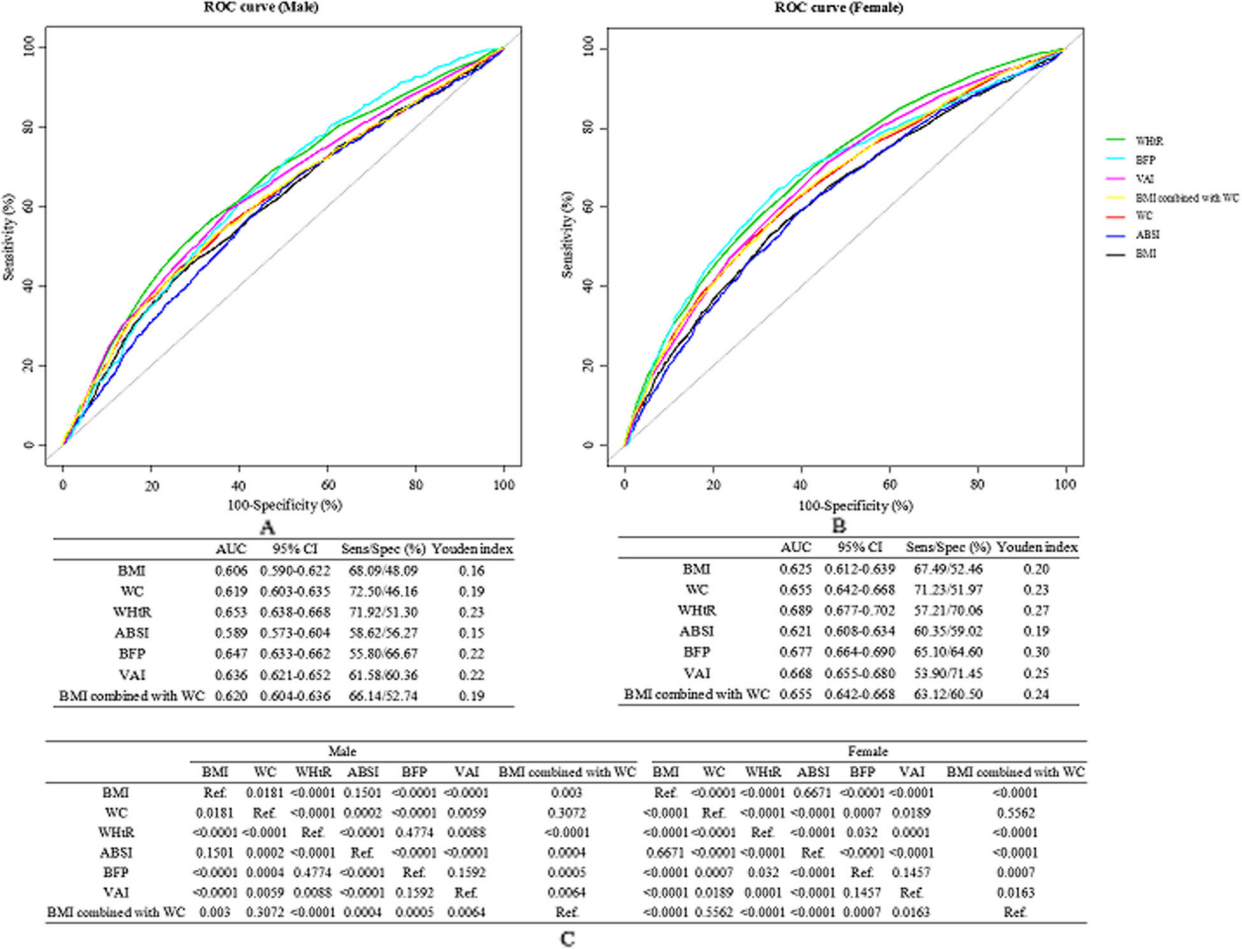
ROC curves of adiposity indices and the combination model including BMI and WC for identifying hypertension according to sex. **a** and **b** ROC curves for the relationships between adiposity indices and hypertension in males and in females, respectively. **c***P* values for pairwise comparison of ROC curves for different adiposity indices in males and in females

**The** ROC curves of adiposity indices and the combination model including BMI and WC for identifying hypertension according to sex were shown in Fig. 2. Figure 2a and Fig. 2b showed the AUCs (95%CI), sensitivity, specificity and Youden’s index of adiposity indices for identifying hypertension by sex. Figure 2c presented *P* values for pairwise comparison of AUCs of different adiposity indices in males and females. The AUCs for WHtR, BFP and VAI were significantly larger than those for other adiposity indices in both males and females (all *P* < 0.01). For males, no statistically significant difference was found in AUCs among WHtR and BFP (0.653 vs. 0.647, *P* = 0.4774). The AUC of WHtR was significantly higher than VAI (0.653 vs. 0.636, *P* < 0.01). For females, the AUCs demonstrated that WHtR was significantly more powerful than BFP and VAI (both *P* < 0.05) for predicting hypertension [WHtR, 0.689 (0.677–0.702); BFP, 0.677 (0.664–0.690); VAI, 0.668 (0.655–0.680)]. Whereas no significant differences were found in AUCs for hypertension among BFP and VAI in both sexes (all *P* > 0.1). The similar results were observed among participants without taking antihypertensive medications (Figure. S1).

The AUCs and 95% CI of adiposity indices for identifying hypertension by sex and age were shown in Table 4. The AUCs for hypertension associated with each adiposity index declined with age in both males and females. For subjects aged < 65 years, WHtR still had the largest AUC. However, for participants aged ≥65 years, BMI had the largest AUC.

**Table 4:** AUC and 95% CI of adiposity indices for identifying hypertension by sex and age

	Age groups (years)		
	15–44	45–64	≥65
Male	164 (1827)^a^	673 (2194)^a^	972 (1940)^a^
BMI	0.756 (0.716, 0.795)	0.638 (0.613, 0.664)	0.614 (0.589, 0.639)
WC	0.749 (0.709, 0.789)	0.651 (0.626, 0.677)	0.597 (0.572, 0.622)
WHtR	0.758 (0.720, 0.797)	0.653 (0.627, 0.678)	0.599 (0.574, 0.624)
ABSI	0.575 (0.532, 0.619)	0.567 (0.541, 0.593)	0.507 (0.481, 0.533)
BFP	0.673 (0.634, 0.713)	0.606 (0.581, 0.631)	0.595 (0.569, 0.620)
VAI	0.727 (0.684, 0.769)	0.625 (0.599, 0.651)	0.607 (0.582, 0.632)
BMI combined with WC	0.756 (0.716, 0.796)	0.652 (0.627, 0.678)	0.605 (0.580, 0.630)
Female	113 (2608)^a^	1016 (3581)^a^	1309 (2423)^a^
BMI	0.738 (0.688, 0.787)	0.619 (0.599, 0.639)	0.612 (0.590, 0.634)
WC	0.718 (0.665, 0.771)	0.619 (0.598, 0.640)	0.604 (0.581, 0.626)
WHtR	0.746 (0.696, 0.795)	0.624 (0.603, 0.644)	0.610 (0.588, 0.632)
ABSI	0.581 (0.527, 0.635)	0.543 (0.522, 0.564)	0.523 (0.500, 0.547)
BFP	0.722 (0.665, 0.778)	0.607 (0.586, 0.628)	0.591 (0.568, 0.613)
VAI	0.725 (0.676, 0.774)	0.616 (0.596, 0.637)	0.602 (0.580, 0.624)
BMI combined with WC	0.727 (0.675, 0.780)	0.622 (0.602, 0.643)	0.610 (0.588, 0.632)

^a^Denotes the number of cases (subjects) for each group

## Discussion

In the population-based cross-sectional study, we explored the associations between different adiposity indices and hypertension by age and sex. The main findings of our present study indicated that anthropometric indices (BMI, WC, WHtR and ABSI) and bioelectrical indices (VAI and BFP) were positively and significantly associated with the prevalence of hypertension in a dose response fashion. Moreover, WHtR was the surrogate obesity marker of predicting hypertension, followed by BFP and VAI, especially in younger (15–44 and 45–64 years) males and females. Our results discourage the use of the BMI.

Previous studies reported that obesity was closely related with hypertension, which is consistent with our findings [5, 10, 20]. We found that all six adiposity indices were positively and significantly associated with hypertension and the ORs for hypertension increased monotonically with increasing levels of six adiposity indices. Moreover, our study showed that ABSI was less strongly associated with hypertension. However, several reports have yielded some conflicting results. ABSI was designed to be minimally associated with weight, height and BMI. Previous studies indicated that ABSI could predicted CVD [21]. Therefore, it had been proposed that ABSI had some potential for being incorporated into clinical guidelines in place of WC and BMI [22]. However, two observational studies reported a modest association between ABSI and risk of hypertension [23, 24]. These inconsistent results suggest that further longitudinal investigations are needed to confirm the association between ABSI and risk of hypertension.

However, the best adiposity index that predicts or associates strongly with hypertension remains controversial and inconclusive. Yang et al. [25] showed that the WHtR was a better predictor than either BMI or WC of metabolic syndrome. Tuan et al. [26] reported that WC and WHtR did not perform better than BMI in predicting hypertension risk among Chinese population aged 18–65 years. Hsu et al. [27] found that BMI was independently associated with elevated BP. Rankinen et al. [28] showed that VAI was the best predictor of obesity. In our study, we found that WHtR was the surrogate obesity marker of predicting hypertension, followed by BFP and VAI. Differences in adipose tissue distribution may contribute to the heterogeneity of clinical and biological manifestations of obesity. There is, however, limited research on the comparison of different adiposity indices in relation to hypertension. We also found that the combined model (BMI + WC) did not increase the predictive power of hypertension. Similar findings have been observed in previous studies [13, 25, 29–31]. These results also provide evidence to support the findings that WHtR, BFP and VAI emerged as the better predictors of hypertension than the traditional obesity indices (BMI and WC). This could be partially explained that visceral rather than sebum fat accumulation was associated with increased secretion of free fatty acids, hyperinsulinemia, insulin resistance, hypertension, and dyslipidemia [32]. WHtR was better than WC because the former considered the height value. Our results discouraged the use of the BMI and the combined model. The major limitation with BMI was that it could not distinguish fat mass from fat-free mass. It may incorrectly estimate the risk of obesity-related diseases in subjects with heavy muscle mass. However, BMI was still recommended still recommends as a universal criterion of overweight and obesity by the World Health Organization. Therefore, future prospective studies with a larger population can further validate the usefulness, as well as the limitations, of WHtR as a marker for risk stratification.

Additionally, we found that the associations between adiposity indices and hypertension varied from age and sex. The AUCs of adiposity indices for identifying hypertension tended to decrease with age in both sexes. This could be partially explained by the less modifiable risk factors (such as metabolic equivalent, smoking, drinking and so on) on the development of hypertension in younger individuals than in older ones [10]. WHtR was the surrogate obesity marker of predicting hypertension in young-aged (15–44 years) subjects and BMI was the surrogate obesity marker of predicting hypertension in elderly (≥ 65 years) participants, which was consistent with the findings in a study by Jiang et al. [10] It suggests that BMI could represents the better predictor of identifying hypertension among elderly participants [27]. The differences between younger and elderly individuals might matters in free fatty acids, secretion of angiotensinogen and sympathetic nervous system activation.

To our knowledge, this was the first study to comparatively assess six adiposity indices (BMI, WC, WHtR, ABSI, VAI and BFP) with respect to their predictive power of hypertension by age and sex in Chinese population. Moreover, it was performed in a large population with strictly standardized methods and validation procedures. Our study also had some limitations. Above all, as a cross-sectional design, it was less power to infer casual inference on the associations between the different adiposity indices and hypertension. In addition, the study participants was restricted to Chinese population in a single province; thus, the generalizability of the results to other populations remained to be verified. Finally, we did not adjust for other potential confounding factors, such as dietary pattern and biochemical indices (e.g., blood lipids and blood glucose).

## Conclusions

In summary, all six adiposity indices were positively and significantly associated with hypertension in a dose response fashion. WHtR was the best for predicting hypertension, followed by BFP and VAI, especially in younger (15–44 and 45–64 year) males and females. Our results discouraged the use of the BMI. Future prospective studies can further validate the usefulness, as well as the limitations, of WHtR as a marker for risk stratification.

## Supplementary information

**Additional file 1 Figure S1.** ROC curves of adiposity indices and the combination model including BMI and WC for identifying hypertension according to sex among participants without taking antihypertensive medications.

**Additional file 2 Table S1.** Minimal data set. This is the S1_S1 Table. Minimal data set Title. This is the S1 Table. Minimal data set legend.

## Data Availability

All data generated or analyzed during this study are included in this published article and its supplementary information files.

## Abbreviations

ABSI: A body shape index
ACEI: Angiotensin-converting enzyme inhibitors
ARB: Angiotensin II receptor blockers
AUC: Area under the curve
BFP: Body fat percentage
BMI: Body mass index
BMR: Basal metabolism rate
CCB: Calcium channel blockers
CI: Confidence interval
CVD: Cardiovascular disease
DBP: Diastolic blood pressure
OR: Odds ratios
RHR: Rest heart rate
SBP: Systolic blood pressure
VAI: Visceral adipose index
WC: Waist circumference
WHtR: Waist-to-height ratio

## References

[1] GBD 2016 Causes of Death Collaborators (2017). Global, regional, and national age-sex specific mortality for 264 causes of death, 1980–2016: a systematic analysis for the Global Burden of Disease Study 2016. Lancet.

[2] Lu J, Lu Y, Wang X, Li X, Linderman GC, Wu C (2017). Prevalence, awareness, treatment, and control of hypertension in China: data from 1.7 million adults in a population-based screening study (China PEACE Million Persons Project). Lancet.

[3] Wang ZW, Chen Z, Zhang LF, Wang X, Hao G, Zhang ZG (2018). Status of hypertension in China: results from the China hypertension survey, 2012-2015. Circulation.

[4] Hu Lihua, Huang Xiao, You Chunjiao, Bao Huihui, Zhou Wei, Li Juxiang, Li Ping, Wu Yanqing, Wu Qinghua, Wang Zengwu, Gao Runlin, Liang Qian, Cheng Xiaoshu (2018). Relationship of sleep duration on workdays and non-workdays with blood pressure components in Chinese hypertensive patients. Clinical and Experimental Hypertension.

[5] Hu L, Huang X, You C, Li JX, Hong K, Li P (2017). Prevalence and risk factors of prehypertension and hypertension in southern China. PLoS One.

[6] Williams B, Mancia G, Spiering W, Rosei EA, Azizi M, Burnier M (2018). 2018 ESC/ESH guidelines for the management of arterial hypertension. Eur Heart J.

[7] Zhang C, Rexrode KM, van Dam RM, Li TY, Hu FB (2008). Abdominal obesity and the risk of all-cause, cardiovascular, and cancer mortality: sixteen years of follow-up in US women. Circulation.

[8] Hall JE, Do CJ, Da SA, Wang Z, Hall ME (2015). Obesity-induced hypertension: interaction of neurohumoral and renal mechanisms. Circ Res.

[9] Schneider HJ, Friedrich N, Klotsche J, Pieper L, Nauck M, John U (2010). The predictive value of different measures of obesity for incident cardiovascular events and mortality. J Clin Endocrinol Metab.

[10] Jiang JC, Deng SY, Chen Y, Liang SY, Ma N, Xu YJ (2016). Comparison of visceral and body fat indices and anthropometric measures in relation to untreated hypertension by age and gender among Chinese. Int J Cardiol.

[11] Neeland IJ, Turer AT, Ayers CR, Powell-Wiley TM, Vega GL, Farzaneh-Far R (2012). Dysfunctional adiposity and the risk of prediabetes and type 2 diabetes in obese adults. JAMA.

[12] Ashwell M, Lejeune S, Mcpherson K (1996). Ratio of waist circumference to height may be better indicator of need for weight management. BMJ.

[13] Ashwell M, Gunn P, Gibson S (2012). Waist-to-height ratio is a better screening tool than waist circumference and BMI for adult cardiometabolic risk factors: systematic review and meta-analysis. Obes Rev.

[14] Krakauer NY, Krakauer JC (2012). A new body shape index predicts mortality hazard independently of body mass index. PLoS One.

[15] Amato MC, Giordano C (2014). Visceral adiposity index: an indicator of adipose tissue dysfunction. Int J Endocrinol.

[16] Chandra A, Neeland IJ, Berry JD, Ayers CR, Rohatgi A, Das SR (2014). The relationship of body mass and fat distribution with incident hypertension: observations from the Dallas heart study. J Am Coll Cardiol.

[17] Wang ZW, Zhang LF, Chen Z, Wang X, Shao L, Guo M (2014). Survey on prevalence of hypertension in China: background, aim, method and design. Int J Cardiol.

[18] Ls L (2011). 2010 Chinese guidelines for the management of hypertension. Zhonghua Xin Xue Guan Bing Za Zhi.

[19] Delong ER, Delong DM, Clarke-Pearson DL (1988). Comparing the areas under two or more correlated receiver operating characteristic curves: a nonparametric approach. Biometrics.

[20] Wang Shuaibing, Peng Rui, Liang Shuying, Dong Kaiyan, Nie Wei, Yang Qian, Ma Nan, Zhang Jianying, Wang Kaijuan, Song Chunhua (2018). Comparison of adiposity indices in relation to prehypertension by age and gender: A community-based survey in Henan, China. Clinical Cardiology.

[21] Bozorgmanesh M, Sardarinia M, Hajsheikholeslami F, Azizi F, Hadaegh F (2016). CVD-predictive performances of "a body shape index" versus simple anthropometric measures: Tehran lipid and glucose study. Eur J Nutr.

[22] Ahima RS, Lazar MA (2013). Physiology. The health risk of obesity--better metrics imperative. Science.

[23] Cheung YB (2014). "a body shape index" in middle-age and older Indonesian population: scaling exponents and association with incident hypertension. PLoS One.

[24] Maessen MF, Eijsvogels TM, Verheggen RJ, Hopman MT, Verbeek AL, de Vegt F (2014). Entering a new era of body indices: the feasibility of a body shape index and body roundness index to identify cardiovascular health status. PLoS One.

[25] Yang H, Xin Z, Feng JP, Yang JK (2017). Waist-to-height ratio is better than body mass index and waist circumference as a screening criterion for metabolic syndrome in Han Chinese adults. Medicine (Baltimore).

[26] Tuan NT, Adair LS, Stevens J, Popkin BM (2010). Prediction of hypertension by different anthropometric indices in adults: the change in estimate approach. Public Health Nutr.

[27] Hsu CH, Lin JD, Hsieh CH, Lau SC, Chiang WY, Chen YL (2014). Adiposity measurements in association with metabolic syndrome in older men have different clinical implications. Nutr Res.

[28] Rankinen T, Kim SY, Perusse L, Després JP, Bouchard C (1999). The prediction of abdominal visceral fat level from body composition and anthropometry: ROC analysis. Int J Obes Relat Metab Disord.

[29] Tseng CH, Chong CK, Chan TT, Bai CH, You SL, Chiou HY (2010). Optimal anthropometric factor cutoffs for hyperglycemia, hypertension and dyslipidemia for the Taiwanese population. Atherosclerosis.

[30] Park SH, Choi SJ, Lee KS, Park HY (2009). Waist circumference and waist-to-height ratio as predictors of cardiovascular disease risk in Korean adults. Circ J.

[31] Zhang ZP, Shi D, Zhang Q, Wang S, Liu K, Meng QT (2018). Visceral adiposity index (VAI), a powerful predictor of incident hypertension in prehypertensives. Intern Emerg Med.

[32] Krakoff LR (2014). Adiposity and risk for hypertension: does location matter?. J Am Coll Cardiol.

