# Supplementary figures and images for: Different adiposity indices and their associations with hypertension among Chinese population from Jiangxi province
Source: BMC Cardiovasc Disord. 2020 Mar 5;20:115. doi: 10.1186/s12872-020-01388-2 (PMC7059680; doi:10.1186/s12872-020-01388-2)

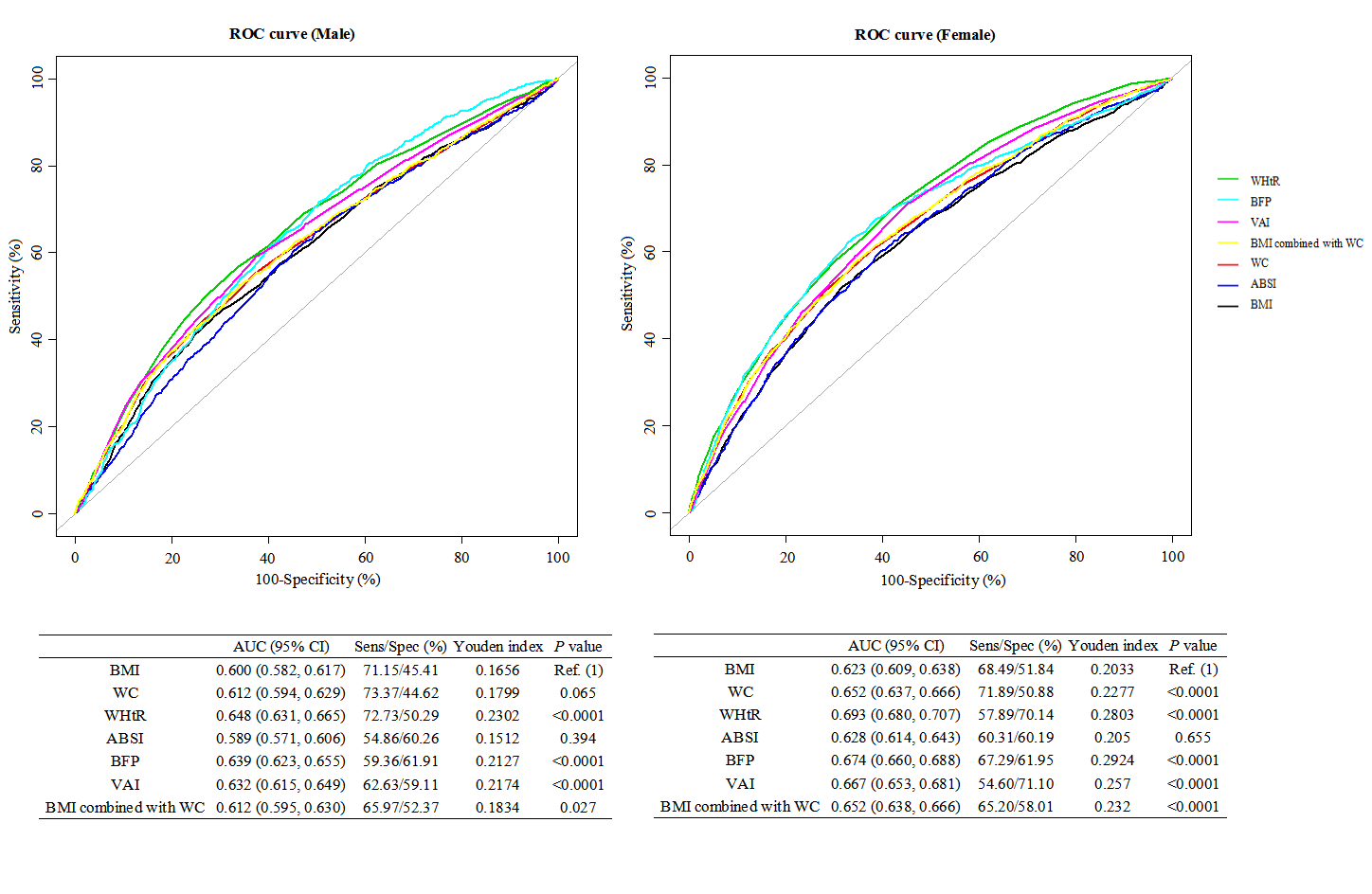

Supplement: Supplementary file 1 — Additional file 1 Figure S1. ROC curves of adiposity indices and the combination model including BMI and WC for identifying hypertension according to sex among participants without taking antihypertensive medications. [file 12872_2020_1388_MOESM1_ESM.tif]
